# Chondroprotective Effect of Cynaroside in IL-1*β*-Induced Primary Rat Chondrocytes and Organ Explants via NF-*κ*B and MAPK Signaling Inhibition

**DOI:** 10.1155/2020/9358080

**Published:** 2020-01-24

**Authors:** Seul Ah. Lee, Bo-Ram Park, Sung-Min Moon, Joon Ho Hong, Do Kyung Kim, Chun Sung Kim

**Affiliations:** ^1^Department of Oral Biochemistry, College of Dentistry, Chosun University, 309 Pilmun-daero, Dong-gu, Gwangju 61452, Republic of Korea; ^2^Department of Dental Hygiene, College of Health and Welfare, Kyungwoon University, 730, Gangdong-ro, Gyeongsangbuk-do 39160, Republic of Korea; ^3^CStech Research Institute, 38 Chumdanventuresoro, Gwangju 61007, Republic of Korea; ^4^Nano Bio Research Center, Jeonnam Bioindustry Foundation, 123, Samtae-ro, Nam-myunm Jangseong-gun, Jeollanam-do 57248, Republic of Korea; ^5^Oral Biology Research Institute, College of Dentistry, Chosun University, 309 Pilmun-daero, Dong-gu, Gwangju 61452, Republic of Korea

## Abstract

Osteoarthritis (OA) is a degenerative joint disease characterized by cartilage degradation and inflammation. Interleukin-1*β* is the key player in the pathogenesis of OA, which induces the expression of various catabolic factors that contribute to cartilage degradation. Cynaroside (luteolin-7-O-glucoside or luteoloside) is a flavonoid that has various pharmacological properties, such as antitumor, anti-inflammatory, and antioxidant activities. In this study, we investigated the chondroprotective effects of cynaroside on IL-1*β*-stimulated chondrocytes and organ explants. The production of nitrite, PGE_2_, collagen type II, and aggrecan was measured by a Griess reagent and ELISAs, and the production of ROS was measured by H_2_DCF-DA fluorescence. The protein levels of iNOS, Cox-2, MMP-1, MMP-3, MMP-13, ADAMTS-4, MAPKs, and the NF-*κ*B p65 subunit were measured by western blot. Proteoglycan analysis was performed by Alcian Blue staining (*in vitro*) and Safranin O staining (*ex vivo*). Cynaroside inhibited IL-1*β*-induced expression of catabolic factors (nitrite, iNOS, ROS, PGE_2_, Cox-2, MMP-1, MMP-3, MMP-13, and ADAMTS-4) and degradation of anabolic factors (collagen type II and aggrecan). Furthermore, cynaroside suppressed IL-1*β*-induced phosphorylation of MAPKs and translocation of the NF-*κ*B p65 subunit into the nucleus. Collectively, these results suggest that cynaroside may be a potential candidate for the development of new therapeutic drugs for the alleviation of OA progression.

## 1. Introduction

Flavonoids occur naturally in the plant kingdom and are usually present in the form of conjugates with the flavonoid aglycone, which exhibits antioxidant properties [1, 2]. Cynaroside (luteolin-7-O-glucoside or luteoloside) is a flavonoid-like compound which is primarily hydrolyzed to luteolin, a flavonoid aglycone in the gastrointestinal tract absorbed into the systemic circulation [1, 3, 4]. In general, it is known that flavonoid aglycones are absorbed more rapidly in greater amounts than their glycosides [5]. However, several counter-studies have reported that with time, the concentrations of cynaroside (10 mg/kg, i.v.) and luteolin (10 mg/kg, i.v.) in rat plasma were within an error range (area under the concentration curves; 229 ± 15 and 261 ± 33 *μ*g min/mL, respectively), and there were no differences in the antioxidant efficacy [1, 4]. In addition, these studies reported that glycosides could be partly absorbed without *β*-glucosidase hydrolysis and there is no difference in their bioavailability [1, 6]. Several studies have reported that cynaroside exhibits various pharmacological activities, such as antitumor [7], anti-inflammation [8], antidiabetic [9], and antioxidant effects [10]. Furthermore, in our previous studies, cynaroside has been reported to be one of the major compounds exhibiting anti-inflammatory and chondroprotective effects in AE-ASL (aqueous extract of *Anthriscus sylvestris* leaves) [11], but the chondroprotective effect of cynaroside has not yet been reported.

Osteoarthritis (OA) is the most prevalent musculoskeletal disorder that affects more than 50% of people over 65 years of age, ultimately hindering the healthy aging life [12]. OA is characterized by an irreversible and progressive loss of articular cartilage, subchondral bone thickening, and synovial inflammation and is accompanied by pain and immobility [12, 13]. Currently, conservative treatment options for OA include non-pharmacological management including diet and physical activity, medications such as analgesics and anti-inflammatory drugs, intra-articular injection of hyaluronan, and joint replacement surgery in the late phases [14, 15]. However, these drugs are temporary and do not relieve or halt the development of OA, and in case of surgery, the risks and the economic burden need to be considered [13, 14]. Therefore, there is an urgent need to search for new potential OA drugs that can relieve, delay, or reverse the development of OA.

The main hallmark of OA is progressive and excessive degradation of cartilage extracellular matrix (ECM), which accounts for 95% of total cartilage tissue mass [16–18]. The ECM is mainly composed of collagen type II (COL2A1) and proteoglycans, such as aggrecan, that provide a high degree of structural integrity to the cartilage and absorb compressive force and impact [19, 20]. They are synthesized and maintained in equilibrium between the anabolism and catabolism of chondrocytes, which are the only cell types present in the cartilage [21]. Therefore, protecting chondrocytes from inflammation may make it possible to continuously maintain a dense ECM, which can be a key strategy for palliation or halting OA.

Interleukin-1 beta (IL-1*β*), a proinflammatory cytokine, is the key player in the development of OA. IL-1*β* accelerates OA by inducing the upregulation of cartilage matrix-degrading enzymes, such as matrix metalloproteinases (MMPs), a disintegrin and metalloproteinase with thrombospondin motifs (ADAMTSs), and other catabolic factors including inflammatory mediators, nitrite oxide (NO), and prostaglandin E_2_ (PGE_2_) [21, 22]. In addition, decomposition products of ECM by these cartilage-degrading enzymes activate synoviocytes, which in turn induce release of these catabolic factors leading to articular cartilage fibrillation, fissures, and erosion in the outer layers [23, 24]. These repetitive cycles of inflammation and catabolism impair the homeostasis of chondrocytes and promote irreversible cartilage matrix degradation leading to OA. Therefore, in the present study, we aimed to determine whether cynaroside has a chondroprotective effect *in vitro* and *ex vivo*, and if so, to investigate the underlying mechanisms of its action.

## 2. Methods

### 2.1. Reagents

IL-1*β* was purchased from ProSpec protein specialists (Rehovot, Israel). Sulfanilamide, N-(1-naphthyl)ethylenediamine dihydrochloride, phosphoric acid, 2,7-dichlorodihydrofluorescein diacetate (H_2_DCFDA), casein, Alcian Blue 8GX, and 3-(4,5-dimethylthiazol-2-yl)-2,5-diphenyltetrazolium bromide (MTT) were purchased form Sigma-Aldrich (St. Louis, MO, USA). The aggrecan ELISA kit and collagen type II ELISA kit were purchased from MyBioSource, Inc. (San Diego, CA, USA) and the PGE_2_ ELISA kit was purchased from R&D Systems (Minneapolis, MN, USA). Dulbecco's modified Eagle's medium/nutrient mixture F-12 (DMEM/F12) and penicillin-streptomycin solution were purchased from WELGENE (Daegu, Republic of Korea). Fetal bovine serum (FBS) was purchased from iNtRON Biotechnology (Gyeonggido, Republic of Korea), and collagenase type II was purchased from Worthington Biochemical Corporation (Lakewood, NJ, USA).

### 2.2. Primary Rat Chondrocyte Culture and Organ Explants

Articular cartilages were isolated from the femoral condyle and the tibial plateau of 5-day postnatal Sprague-Dawley (SD) rats, and the tissues were enzymatically digested with 0.2% (*w*/*v*) type II collagenase dissolved in DMEM/F12 for 45 min at 37°C, repeated twice. Afterwards, the cartilage pieces were digested with 0.2% (*w*/*v*) collagenase, type II overnight at 37°C. Undissociated cells and debris were filtered through a cell strainer (0.45 *μ*m), and separated articular chondrocytes were seeded directly into 6- or 12-well cell culture plates at the density of 1 × 10^6^ cells/mL. For organ culture, the legs of 2-week postnatal SD rats were cut out; the skin was removed, washed thrice with 1x PBS containing 2% antibiotics, and randomly assigned to 15 mL conical tubes. The cells and explant organ were cultured in DMEM/F12 containing 10% FBS and 1% antibiotic (penicillin and streptomycin) at 37°C with 5% CO_2_ for 3 days. The culture medium was replaced every other day. The chondrocytes were not passaged to avoid phenotype loss and dedifferentiation. When the cell confluence reached 80–90% after three days, the culture medium was exchanged with starvation medium (DMEM/F12+1% FBS+1% antibiotic), and samples were processed after overnight incubation. To investigate the chondroprotective effect of cynaroside, primary rat chondrocytes and organ explants were pretreated with 0.1% dimethyl sulfoxide (DMSO, control) or various concentrations of cynaroside for 1 h and stimulated with IL-1*β* (10 ng/mL) for 24 h or 4 days, respectively. Animal procedures were approved by the Chosun University Institutional Animal Care and Use Committee (CIACUC2018-S0046).

### 2.3. Cell Viability

The cytotoxicity of cynaroside on chondrocytes was measured using MTT assay, according to the manufacturer's protocol. In brief, primary rat chondrocytes were cultured for 3 days in 12-well plates at the density of 1 × 10^6^ cells/mL and incubated in different concentrations of cynaroside (0, 40, 80, and 160 *μ*M) for 24 h. Post incubation, MTT solution (5 mg/mL) was added to each well (100 *μ*L/well), and cells were incubated for another 4 h at 37°C. After removing the cell culture medium containing the MTT solution, DMSO was added to each well (1 mL/well) and absorbance was measured at 590 nm using a microplate reader (Epoch BioTek Instruments Inc., Winooski, VT, USA). Experiments were performed five times.

### 2.4. Measurement of Reactive Nitrogen Species (RNS)

The nitrite accumulation in the culture medium was measured by a Griess reagent. Primary rat chondrocytes were pretreated with cynaroside for 1 h and stimulated with IL-1*β* (10 ng/mL) for 24 h. Culture medium (100 *μ*L) was mixed with 100 *μ*L of the Griess reagent (1% (*w*/*v*) sulfanilamide and 0.1% (*w*/*v*) naphthylethylenediamine in 5% (*v*/*v*) phosphoric acid). The absorbance was measured at 540 nm using a microplate reader (Epoch BioTek Instruments Inc.). Nitrite was quantified based on a sodium nitrite standard curve.

### 2.5. Measurement of Reactive Oxygen Species (ROS)

IL-1*β*-induced ROS levels were quantified by measuring the conversion of fluorescent DCF (dichlorofluorescein) from nonfluorescent H_2_DCF-DA (2′,7′-dichlorofluorescein diacetate). Primary rat chondrocytes were seeded into a clear-bottom, black 96-well plate at 5 × 10^5^ cells/mL and incubated for three days till cells reached 80–90% confluence. Cells were pretreated with cynaroside for 1 h and stimulated with IL-1*β* (10 ng/mL) for 24 h. Culture medium was replaced with Hank's Balanced Salt Solution (HBSS) containing H_2_DCF-DA (20 *μ*M), and cells were incubated for 30 min. Intracellular ROS levels were measured at 485 nm/535 nm (excitation/emission) in a fluorescence microplate reader (Tecan Infinite F200, Tecan, Grodig, Austria).

### 2.6. Measurement of PGE_2_, TNF-*α*, Collagen Type II, and Aggrecan

The concentrations of PGE_2_, TNF-*α*, collagen, type II, and aggrecan in the culture medium or cells were measured using commercial ELISA kits (PGE_2_ and TNF-*α*, R&D Systems; collagen type II and aggrecan, MyBioSource) according to the manufacturer's protocol. All assays were performed in triplicate.

### 2.7. Western Blot Analysis

Primary rat chondrocytes were pretreated with cynaroside for 1 h and stimulated with IL-1*β* (10 ng/mL) for 1 h or 24 h. Harvested cells were washed twice with ice-cold PBS and lysed using PRO-PREP protein extraction solution (iNtRON Biotechnology) to extract whole intracellular proteins. Cytoplasmic and nuclear proteins were extracted using NE-PER™ Nuclear and Cytoplasmic Extraction Reagents (Thermo Fisher Scientific, IL, USA) according to the manufacturer's protocol. After harvesting, the articular cartilage was sliced from the explant organ using a blade, and proteins from the articular cartilage slice were extracted using a PRO-PREP protein extraction solution. Cartilage slices containing lysis buffer were homogenized, incubated for 30 min on ice, and centrifuged at 14,000 × *g* for 15 min at 4°C. Protein concentrations in each lysate were quantified using the Bicinchoninic Acid Protein Assay Kit (Pierce, Rockford, IL, USA). Equivalent amounts (20 *μ*g) of lysate protein were separated on 8 or 15% SDS-polyacrylamide gel and transferred to a polyvinylidene difluoride membrane (Bio-Rad Laboratories, Hercules, CA, USA). The transblotted membranes were blocked with 5% bovine serum albumin at room temperature for 30 min, followed by overnight incubation with primary antibodies (1 : 1000, except *α*-tubulin; 1 : 5000) at 4°C. The membranes were then washed thrice with Tris-buffered saline containing 0.1% Tween-20 (TBST) and incubated with horseradish peroxidase-linked secondary antibody (1 : 2500) at room temperature for 1 h. Protein bands were detected using an enhanced chemiluminescence (ECL) kit (Millipore, Bedford, MA, USA) and visualized using a MicroChemi 4.2 imager (DNR Bioimaging Systems, Jerusalem, Israel).

### 2.8. Casein Zymography

A casein-substrate zymography was carried out using culture supernatants from chondrocytes pretreated with cynaroside for 1 h and stimulated with IL-1*β* (10 ng/mL) for 24 h. Samples mixed with nonreducing buffer were electrophoresed at 4°C on an 8% SDS-PAGE gel containing copolymerized casein. After electrophoresis, gels were rinsed with 2.5% (*v*/*v*) Triton X-100 for 30 min with gentle shaking and were incubated with a zymogram renaturing buffer (10 mM CaCl_2_, 50 mM Tris-HCl (pH 6), and 0.15 M NaCl) at 37°C for 48 h. Finally, gels were stained with 0.1% (*w*/*v*) Coomassie Brilliant Blue R-250 for 20 min, followed by destaining and imaging the gels by photography. Zone of clearance in bands indicated casein degradation.

### 2.9. Alcian Blue Stain

Primary rat chondrocytes were cultured in 6-well cell culture plates at the density of 1 × 10^6^ cells/mL. The cells were pretreated with cynaroside for 1 h and stimulated with IL-1*β* (10 ng/mL) for 48 h. The cells were fixed with 70% ethanol for 20 min and stained with 0.1% Alcian Blue 8GX in 0.1 N HCl at room temperature overnight. The cells were photographed after washes with 1x PBS to remove unstained cells. After that, the dye was dissolved in 6 M guanidine-HCl, and the absorbance was measured at 650 nm using a microplate reader (Epoch BioTek Instruments).

### 2.10. Histology Analysis and Stain

Organ explants were fixed in 10% neutral buffered formalin for 1 day at 4°C, decalcified with 0.5 M EDTA (pH 7.4) for 3 days at 4°C, dehydrated through a series of ethanol solutions, and embedded in paraffin blocks. The explants were then sectioned at 10 *μ*m thickness and stained with Safranin O/Fast Green. The stained sections were digitally photographed under a microscope (Leica Camera AG, Wetzlar, Germany). The stained sections were scored based on the Osteoarthritis Research Society International (OARSI) advanced Osteoarthritis Cartilage Histopathology Assessment System (0-6.5) and used a summed OARSI score to evaluate the degree of articular cartilage destruction.

### 2.11. Statistical Analyses

All data were obtained from at least three independent experiments. The results are expressed as the mean ± standard deviation (SD). A one-way analysis of variance followed by Tukey's test was used for multigroup comparisons using the GraphPad Prism 5.0 (GraphPad Software Inc., CA, USA). A *p* value <0.05 was considered significant.

## 3. Results

### 3.1. Effect of Cynaroside on Viability of Rat Primary Chondrocytes

**Figure 1:**
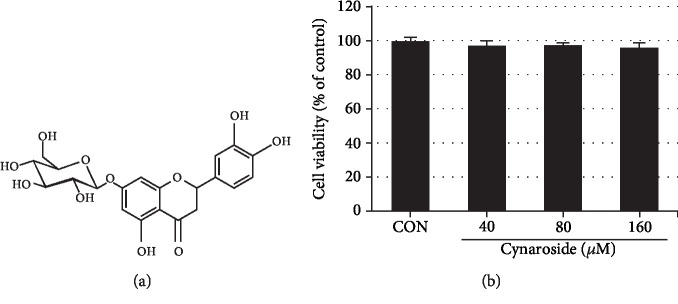
Effects of cynaroside on chondrocyte viability. (a) Chemical formula of cynaroside. (b) Cells were treated with cynaroside (40, 80, and 160 *μ*M) for 24 h, and viability was determined by MTT assay. Cells incubated without cynaroside were used as controls and were considered 100% viable. Data are represented as mean ± SD of three independent experiments. CON: control.

To evaluate the cytotoxicity of cynaroside, chondrocytes were treated with varying concentrations of cynaroside (0, 40, 80, and 160 *μ*M) for 24 h and MTT assay was performed. As shown in Figure 1, cynaroside treatment had no cytotoxic effect when compared to the control up to 160 *μ*M.

### 3.2. Cynaroside Inhibits IL-1*β*-Induced Expression of Oxidative, Nitrosative, and Proinflammatory Mediators and TNF-*α* in Rat Primary Chondrocytes

**Figure 2:**
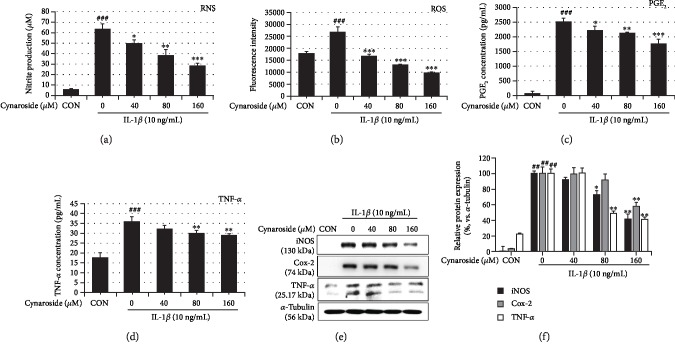
Inhibitory effects of cynaroside on IL-1*β*-induced nitrite, ROS, PGE_2_, TNF-*α*, iNOS, and Cox-2 in primary rat chondrocytes. Cells were pretreated with cynaroside (40, 80, and 160 *μ*M) for 1 h, followed by IL-1*β* (10 ng/mL) stimulation for 24 h. (a) Nitrite production was determined in the cultured medium using a Griess reagent. (b) ROS levels were detected by H_2_DCF-DA probe. (c, d) PGE_2_ and TNF-*α* production was determined in the cultured medium by ELISA. (e) Expression of the iNOS, Cox-2, and TNF-*α* was determined using western blot analysis. (f) Quantitative data of (e) were analyzed using ImageJ software. *α*-Tubulin served as an internal control. Expression results are represented as mean ± SD of three independent experiments. ^##^*p* < 0.01 compared with the control group; ^∗^*p* < 0.05, ^∗∗^*p* < 0.01, and ^∗∗∗^*p* < 0.001 compared with the IL-1*β*-treated group. CON: control; ROS: reactive oxygen species; PGE_2_: prostaglandin E_2_; TNF-*α*: tumor necrosis factor-alpha; iNOS: inducible nitrite oxide; Cox-2: cyclooxygenase-2.

Persistent and excessive inflammation and oxidative stress are well known as the main causes of OA induction and acceleration due to their cartilage matrix-degrading ability [21]. Therefore, we first assessed the effect of cynaroside on IL-1*β*-induced production of nitrite, ROS, PGE_2_, and TNF-*α* in rat primary chondrocytes using the Griess reagent, H_2_DCF-DA, and ELISA, respectively. As shown in Figures 2(a)–2(d), IL-1*β* significantly induced the production of nitrite (11-fold, 63.34 ± 4.98 *μ*M), ROS (2.3-fold, 26,967.67 ± 1939.79), PGE_2_ (50-fold, 2495.74 ± 135.85 pg/mL), and TNF-*α* (twofold, 35.9 ± 2.58 pg/mL), compared to that in the control (5.41 ± 1.24 *μ*M, 11,724.33 ± 194.31, 49.756 ± 87.35 pg/mL, and 17.73 ± 2.11 pg/mL, respectively). However, pretreatment with cynaroside suppressed the induction of nitrite, ROS, PGE_2_, and TNF-*α* in a dose-dependent manner and showed inhibition rates of 54%, 64%, 29%, and 20%, respectively, at 160 *μ*M. Further, to verify the inhibitory effect of cynaroside on IL-1*β*-induced production of nitrite and PGE_2_, we examined the expression of iNOS and Cox-2 by western blotting. As shown in Figure 2(e), we noted that pretreatment with cynaroside suppressed IL-1*β*-mediated increase in the expression of the above factors in a dose-dependent manner. In addition, pretreatment with cynaroside inhibited IL-1*β*-induced expression of TNF-*α* (Figures 2(d) and 2(e)). These results suggest that cynaroside has a potent anti-inflammatory effect on IL-1*β*-induced inflammatory response in rat primary chondrocytes.

### 3.3. Cynaroside Inhibits IL-1*β*-Induced Expression of Cartilage Matrix-Degrading Enzymes in Rat Primary Chondrocytes

**Figure 3:**
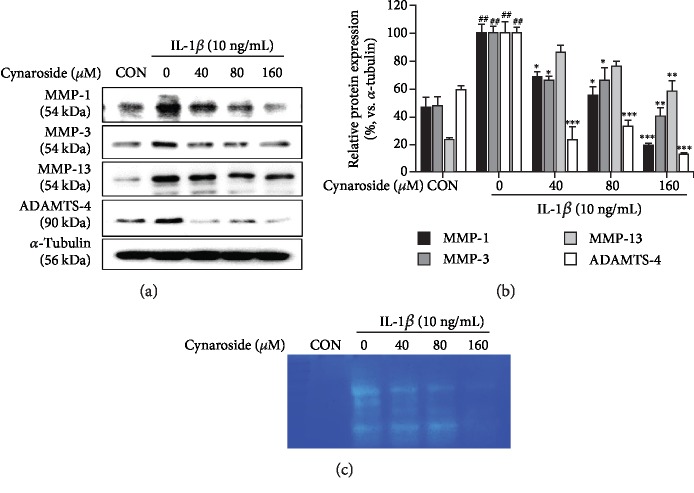
Inhibitory effect of cynaroside on IL-1*β*-induced MMP-1, MMP-3, MMP-13, and ADAMTS-4 in primary rat chondrocytes. Cells were pretreated with cynaroside (40, 80, and 160 *μ*M) for 1 h, followed by IL-1*β* (10 ng/mL) stimulation for 24 h. (a) Protein levels of MMP-1, MMP-3, MMP-13, and ADAMTS-4 were determined using western blot analysis. *α*-Tubulin served as an internal control. (b) Quantitative data of (a) were analyzed using ImageJ software. (c) Activity of MMPs was measured in conditioned medium using casein zymography. ^##^*p* < 0.01 compared with the control group; ^∗^*p* < 0.05, ^∗∗^*p* < 0.01, and ^∗∗∗^*p* < 0.001 compared with the IL-1*β*-treated group. CON: control; MMP: matrix metalloproteinase; ADAMTS-4: a disintegrin and metalloproteinase with thrombospondin motifs 4.

Inflammation mediators, NO and PGE_2_, induced the secretion of MMPs and ADAMTSs, which further degrade collagen and aggrecan, essential constituents of the cartilage matrix [23]. Therefore, we investigated the effect of cynaroside on the IL-1*β*-induced expression of MMP-1, MMP-3, MMP-13, and ADAMTS-4 in rat primary chondrocytes. We observed that protein levels of MMP-1, MMP-3, MMP-13, and ADAMTS-4 increased in IL-1*β*-stimulated chondrocytes compared to the control but were significantly inhibited in cynaroside-pretreated chondrocytes (Figures 3(a) and 3(b)). Furthermore, casein zymography performed to analyze MMP activity showed that IL-1*β* treatment significantly increased the secretion and casein proteolytic activity of these enzymes, but pretreatment with cynaroside suppressed this increase in activity (Figure 3(c)), suggesting that cynaroside suppressed IL-1*β*-induced expression of cartilage-degrading enzymes.

### 3.4. Cynaroside Protects IL-1*β*-Induced Degradation of Collagen Type II and Aggrecan in Rat Primary Chondrocytes

**Figure 4:**
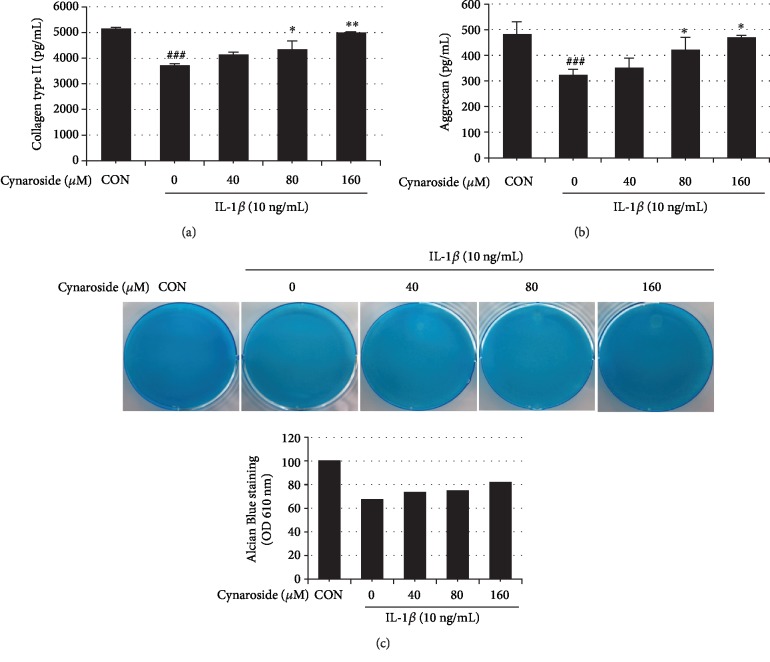
Effect of cynaroside on IL-1*β*-induced collagen type II and aggrecan in primary rat chondrocytes. (a) Cells were pretreated with cynaroside (40, 80, and 160 *μ*M) for 1 h, followed by IL-1*β* (10 ng/mL) stimulation for 24 h. Collagen type II and aggrecan were measured in cultured medium using ELISA. Results are represented as mean ± SD of three independent experiments. ^##^*p* < 0.01 compared with the control group; ^∗^*p* < 0.05 and ^∗∗^*p* < 0.01 compared with the IL-1*β*-treated group. CON: control. (b) Cells were pretreated with cynaroside (40, 80, and 160 *μ*M) for 1 h, followed by IL-1*β* (10 ng/mL) stimulation for 48 h. Proteoglycan contents were determined using Alcian Blue stain.

Next, we investigated the effect of cynaroside on the IL-1*β*-induced degradation of collagen type II and aggrecan in rat primary chondrocytes. Collagen type II and aggrecan amounts in the cells were measured by ELISA, and proteoglycan was analyzed by Alcian Blue staining. As shown in Figures 4(a) and 4(b), IL-1*β* stimulation downregulated collagen type II and aggrecan expression (3730.667 ± 42.42 pg/mL and 324 ± 21.22 pg/mL, respectively) when compared to the control (5144 ± 47.14 pg/mL and 484 ± 49.49 pg/mL, respectively). However, pretreatment with cynaroside significantly prevented IL-1*β*-induced degradation of collagen type II and aggrecan as compared to that observed with IL-1*β* treatment alone (Figures 4(a) and 4(b)). Furthermore, the protective effect of cynaroside on proteoglycan was confirmed by Alcian Blue staining. IL-1*β*-treated samples were weakly stained as compared to the control, whereas pretreatment with cynaroside showed increased proteoglycan staining in a dose-dependent manner (Figure 4(c)). These results suggest that cynaroside has potent cartilage matrix-protective effects by suppressing IL-1*β*-induced degradation of collagen type II and aggrecan in rat primary chondrocytes.

### 3.5. Cynaroside Inhibits IL-1*β*-Induced Activation of NF-*κ*B and MAPKs in Rat Primary Chondrocytes

**Figure 5:**
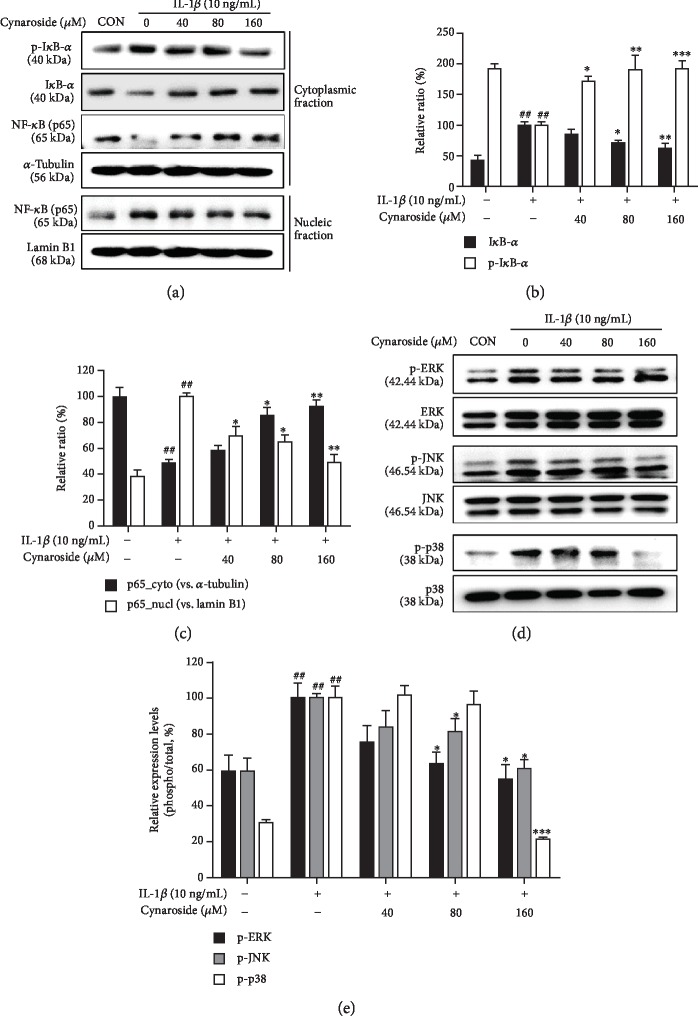
Effects of cynaroside on IL-1*β*-induced activation of NF-*κ*B and phosphorylation of MAPKs in primary rat chondrocytes. Cells were pretreated with cynaroside (40, 80, and 160 *μ*M) for 1 h, followed by IL-1*β* (10 ng/mL) stimulation for 1 h. (a) Phosphorylation levels of I*κ*B-*α* and NF-*κ*B p65 translocation to the nucleus were determined using western blot analysis. (b, c) Quantitative data of (a) were analyzed using ImageJ software. (d) Protein levels of phosphorylation of MAPKs (ERK, JNK, and p38) were determined using western blot analysis. (e) Quantitative data of (d) were analyzed using ImageJ software. *α*-Tubulin and lamin B1 were used as cytosolic and nuclear internal controls, respectively. ^##^*p* < 0.01 compared with the control group; ^∗^*p* < 0.05, ^∗∗^*p* < 0.01, and ^∗∗∗^*p* < 0.001 compared with the IL-1*β*-treated group. CON: control; I*κ*B-*α*: nuclear factor of kappa light polypeptide gene enhancer in B-cell inhibitor, alpha; p65: NF-kappa-B p65 subunit; ERK: extracellular signal-regulated kinase; JNK: c-Jun N-terminal kinase.

Since NF-*κ*B and MAPK pathways are closely associated with the IL-1*β*-mediated expression of cartilage-degrading factors, inhibition of these pathways is generally regarded as one of the important mechanisms for inhibiting expression of cartilage-degrading enzymes [25]. Therefore, we examined the chondroprotective properties of cynaroside by assessing whether cynaroside affects these pathways. As shown in Figure 5(a), IL-1*β* alone induced the phosphorylation and degradation of I*κ*B-*α*, thereby enhancing the nuclear accumulation of NF-*κ*B subunit p65. However, pretreatment with cynaroside inhibited the IL-1*β*-induced nuclear accumulation of NF-*κ*B subunit p65 by suppressing phosphorylation and degradation of I*κ*B-*α*. Furthermore, IL-1*β* treatment induced the phosphorylation of ERK, JNK, and p38 MAPK without affecting their total protein expression levels, but pretreatment with cynaroside significantly inhibited the IL-1*β*-induced phosphorylation of ERK, JNK, and p38 MAPK in a dose-dependent manner (Figure 5(b)). These results suggest that the NF-*κ*B and MAPK pathways may mediate the chondroprotective effect of cynaroside in rat primary chondrocytes.

### 3.6. Chondroprotective Effect of Cynaroside *Ex Vivo*

**Figure 6:**
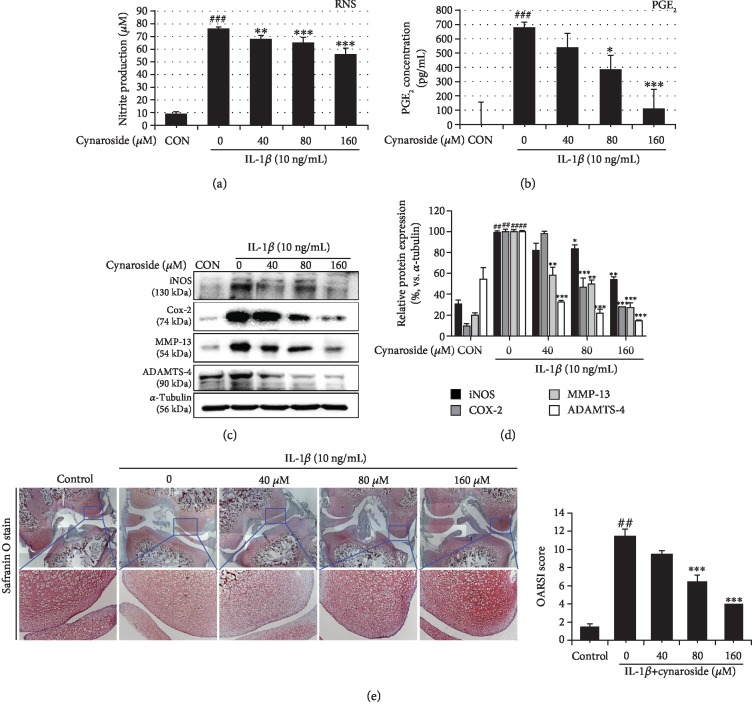
Chondroprotective effects of cynaroside in rat explant organs (legs). Explanted legs were pretreated with cynaroside (40, 80, and 160 *μ*M) for 1 h, followed by IL-1*β* (10 ng/mL) stimulation for 4 days. (a) Nitrite production was determined in the cultured medium using a Griess reagent. (b) PGE_2_ production was determined in the cultured medium using ELISA. (c) Protein levels of iNOS, Cox-2, MMP-13, and ADAMTS-4 were determined using western blot analysis. (d) Quantitative data of (c) were analyzed using ImageJ software. *α*-Tubulin served as an internal control. (e) Histological analysis of proteoglycan loss was carried out by Safranin O staining and the Osteoarthritis Research Society International (OARSI) advanced Osteoarthritis Cartilage Histopathology Assessment System. Results are represented as mean ± SD of three independent experiments. ^##^*p* < 0.01 compared with the control group; ^∗^*p* < 0.05, ^∗∗^*p* < 0.01, and ^∗∗∗^*p* < 0.001 compared with the IL-1*β*-treated group. CON: control; RNS: reactive nitrogen species: PGE_2_: prostaglandin E_2_; iNOS: inducible nitrite oxide; Cox-2: cyclooxygenase-2; MMP: matrix metalloproteinase; ADAMTS-4; a disintegrin and metalloproteinase with thrombospondin motifs 4.

We carried out *ex vivo* culture to confirm the chondroprotective effect of cynaroside in the cartilage tissues. The excised legs were randomly distributed to 15 mL conical tubes and cultured. These explants were then pretreated with 0.1% DMSO (control) or various concentrations of cynaroside for 1 h and stimulated with IL-1*β* (10 ng/mL) for 4 days. As shown in Figures 6(a) and 6(b), IL-1*β* alone significantly induced the production of both nitrite and PGE_2_, but pretreatment with cynaroside inhibited this induction. Pretreatment with cynaroside markedly suppressed the protein expression of IL-1*β*-induced iNOS, Cox-2, MMP-13, and ADAMTS-4, which is consistent with the *in vitro* results (Figures 6(c) and 6(d)). Moreover, Safranin O staining of IL-1*β* was weaker than that of the control, whereas pretreatment with cynaroside was more intense than that of IL-1*β* in a concentration-dependent manner (Figure 6(e)). These results suggest that proteoglycan degradation by IL-1*β* can be protected by cynaroside pretreatment.

## 4. Discussion

OA is a common degenerative joint disease, with a considerable social and economic burden disease owing to the absence of therapeutic drugs [26]. The exact pathogenic mechanism of OA has not yet been elucidated, but it is widely accepted that OA is triggered by degradation of the cartilaginous matrix due to inflammation-induced upregulation of catabolism in chondrocytes [16, 20]. Therefore, a commonly used approach utilizes the inhibition of ECM degradation by protecting the chondrocytes from external stimuli as a key target for OA mitigation [16–20]. In this study, we demonstrate that cynaroside shows beneficial chondroprotective effects by inhibiting the expression of various pathological factors affecting OA, including nitrosative and oxidative stress, degradation of articular ECM, and expression of proinflammatory cytokines and mediators via NF-*κ*B and MAPK signaling pathways.

IL-1*β* is a potent catabolic factor in OA pathogenesis that induces the initiation and progression of ECM degradation in chondrocytes and triggers the expression of other catabolic factors, such as iNOS, NO, COX-2, PGE_2_, TNF-*α*, MMPs, and ADAMTSs, which contribute to chondrocyte dysfunction [21, 27]. Furthermore, as OA patients exhibit relatively high ROS levels and low antioxidant activity, increasing the antioxidant as well as anti-inflammatory activities could also be an effective strategy in OA mitigation [28, 29]. In our study, the production of NO, ROS, PGE_2_, and TNF-*α* and the expression of iNOS, Cox-2, and TNF-*α* increased upon IL-1*β* treatment, but pretreatment with cynaroside rescued this effect. These results are consistent with previous studies that analyzed the ameliorating effect of OA, and pretreatment with wogonin with human chondrocytes and expression of NO, ROS, PGE_2_, and Cox-2 were reduced when treated with IL-1*β* [30].

MMPs are a family of proteolytic enzymes which contributes to OA development by degrading the COL2A1 and proteoglycan that are essential constituents of ECM that protect articular cartilage and chondrocytes [31]. In particular, among these MMPs, inhibition of MMP-13 expression is thought to be very important for OA mitigation due to its ability to robustly degrade COL2A1 and the proteoglycan [31]. ADAMTS-4 is an enzyme that degrades the proteoglycan aggrecan and its activity is deeply involved in OA pathogenesis [32]. In our study, we noted that IL-1*β* treatment increased the expression and activity of MMP-1, MMP-3, MMP-13, and ADAMTS-4. In addition, IL-1*β* treatment induced the degradation of COL2A1 and aggrecan. However, pretreatment with cynaroside prohibited degradation of COL2A1 and aggrecan by suppressing the IL-1*β*-induced activity of MMPs (MMP-1, MMP-3, and MMP-13) and ADAMTS-4. These effects of cynaroside were similar to those of luteolin treatment which suppressed the expression of MMP-1, MMP-3, and MMP-13 [33]. These results clearly suggest that cynaroside has a chondroprotective effect against IL-1*β*-related induction and development of OA.

NF-*κ*B and MAPK pathways are critical signal transduction pathways associated with inflammatory response in OA development [34]. NF-*κ*B exists in an inactive state by binding with I*κ*B in the cytoplasm, but in the presence of IL-1*β*, phosphorylation and degradation of I*κ*B result in the translocation of NF-*κ*B p65 into the nucleus, resulting in induction of expression of inflammatory mediators [35]. In addition, MAPKs play an essential role in chondrogenesis, ERK mediates chondrocyte proliferation and gene expression, and p38 and JNK play an important role in inflammation and destruction of articular cartilage [36, 37]. Therefore, inhibition of these pathways is thought to be crucial in suppressing inflammation, and many studies have reported that inhibition of MAPK (ERK1/2, JNK, and p38) phosphorylation and NF-*κ*B p65 translocation into the nucleus resulted in the inhibition of IL-1*β*-induced MMPs, NO, PGE_2_, and proinflammatory cytokines in rabbit chondrocytes [34]. In our study, cynaroside inhibited the IL-1*β*-induced phosphorylation of MAPKs (ERK1/2, JNK, and p38) and translocation of the NF-*κ*B p65 subunit to the nucleus, suggesting that the chondroprotective effect of cynaroside is at least partially via MAPKs and the NF-*κ*B signaling pathway.

Collectively, pretreatment with cynaroside (especially at a concentration of 160 *μ*M) effectively inhibited IL-1*β*-induced inflammatory factors (NO, ROS, PGE_2_, iNOS, Cox-2, and TNF-*α*) and cartilage-degrading enzymes (MMP-1, MMP-3, MMP-13, and ADAMTS-4). Furthermore, cynaroside also protects collagen type II and aggrecan, which are the constitutive components of chondrocyte ECM, from degradation by IL-1*β* treatment. These results suggest that cynaroside, a major component of AE-ASL (aqueous extract of *Anthriscus sylvestris* leaves), exerts chondroprotective effects in IL-1*β*-induced OA development.

Despite discovering the beneficial effects of cynaroside, this study has some limitations. First, we were not able to elucidate the posttreatment effect of cynaroside after IL-1*β* treatment, which would be an interesting avenue to explore. Second, we used chondrocytes isolated from very young rats and cultured them as per the monolayer method for the *in vitro* study, similar to many other studies. However, OA occurs predominantly in elderly patients; therefore, it would be interesting to use chondrocytes isolated from older rats or isolated from OA patients, which would exhibit more significant changes than chondrocytes isolated from younger rats in terms of growth rates and passage number. In addition, further studies are also needed in special *in vitro* conditions similar to the *in vivo* environment. Third, the luteolin transformation ratio of cynaroside is 48.78 ± 0.12%. Therefore, future studies are needed to compare efficacy with luteolin. For these reason, further research is needed to clarify the chondroprotective effect of cynaroside in OA development, which could make it a potential candidate for the development of new therapeutic drugs.

## Data Availability

The data used to support the findings of this study are available from the corresponding author upon request.
